# Rhabdomyolysis and Sodium-Glucose–Linked Transport Inhibitors in Patients Taking Statins

**DOI:** 10.1001/jamanetworkopen.2024.46641

**Published:** 2024-11-14

**Authors:** Ziv Harel, Nivethika Jeyakumar, Graham Smith, Joel G. Ray, Kristin K. Clemens, David N. Juurlink

**Affiliations:** 1 Division of Nephrology, Department of Medicine, St. Michael’s Hospital, Toronto, Ontario, Canada; 2 ICES, Ontario, Canada; 3 Lawson Health Research Institute, London Health Sciences Centre, London, Canada; 4 Department of Medicine, St. Michael’s Hospital, Toronto, Ontario, Canada; 5 Division of Endocrinology and Metabolism, Department of Epidemiology and Biostatistics Western University, London Ontario, Canada; 6 Centre for Diabetes, Endocrinology and Metabolism, St. Joseph’s Health Care London, London, Ontario, Canada; 7 Department of Medicine, Sunnybrook Hospital, University of Toronto, Toronto, Ontario, Canada

## Abstract

This case-control study investigates the risk of rhabdomyolysis among patients receiving sodium-glucose cotransporter-2 (SGLT2) inhibitors.

## Introduction

Sodium-glucose cotransporter-2 inhibitors (SGLT2is) are increasingly used to treat diabetes, heart failure, and chronic kidney disease.^1^ Approximately 80% of individuals taking SGLT2is also receive statins.^2^ A 2020 case report^3^ described the onset of rhabdomyolysis after initiation of canagliflozin in a patient taking rosuvastatin. Concomitantly high plasma rosuvastatin concentration suggested a drug interaction with canagliflozin.^3^ Our study assessed the risk of rhabdomyolysis upon SGLT2i initiation in individuals receiving chronic statin therapy.

## Methods

This population-based nested case-control study was conducted in Ontario, Canada, July 1, 2015, to September 23, 2020 (eMethods in Supplement 1). This study’s use of data is authorized under section 45 of Ontario’s Personal Health Information Protection Act and approved by the ICES Privacy and Legal Office; the study did not require research ethics board review. Each patient was identified from within several provincial health care administrative databases using a unique encrypted identifier (eTable 1 in Supplement 1). We included adults aged 66 years or older continuously prescribed a statin based on 2 or more statin prescriptions in the 210 days before the index date, with the most recent prescription crossing the index date.

From the statin cohort, cases were individuals who had an emergency department visit or a primary hospitalization diagnosis of rhabdomyolysis. We defined rhabdomyolysis by *International Statistical Classification of Diseases and Related Health Problems, Tenth Revision, with Canadian Enhancements *(*ICD-10-CA*) codes M628 or T796 or a serum creatine kinase value 5 or more times the upper limit of normal, both validated measures of this condition.^4,5^ The date of the rhabdomyolysis event served as the index date for all analyses. Each case was matched to 5 controls by age (plus or minus 1 year), sex, and disease risk score (plus or minus 0.2 SDs). Disease risk score components are described in eTable 2 in Supplement 1. Each control was randomly assigned an index date to mirror the temporal distribution of case index dates.

The exposure of interest and comparator exposure were prescription of an SGLT2i or a dipeptidyl peptidase 4 inhibitor (DPP4i), respectively, within 100 days of receiving a statin prescription. An SGLT2i or DDP4i could be prescribed as a single agent or combined with another diabetes medication. An individual could also have multiple at-risk intervals of SGLT2i or DPP4i use; however, each interval could not be preceded by a prescription for the other drug type or a subclass of each exposure in the 100 days before the index date.

Conditional logistic regression generated adjusted odds ratios (aORs) and 95% CIs for the association between SGLT2i exposure and rhabdomyolysis, adjusting for a frailty index and the number of hospitalizations within 365 days before the index date. A secondary analysis explored the association between type of SGLT2i and rhabdomyolysis. Analyses were performed January to April 2023 at ICES using SAS statistical software version 9.4 (SAS Institute) and followed the STROBE reporting guideline.

## Results

Among 222 187 older adults receiving chronic statin therapy with 1 or more SGLT2i or DDPi prescriptions, there were 2928 cases with rhabdomyolysis and 14 640 matched controls (mean [SD] age of cases and controls, 79.1 [6.8] years; 43.5% female). Among cases and controls, 99.3% had diabetes and 57.3% had chronic kidney disease (Table 1).

**Table 1.** Baseline Study Population Characteristics

Characteristic	Adults, No. (%)		Standardized difference
	Cases (n = 2928)	Controls (n = 14 640)	
Age, mean (SD), y	79.1 (6.8)	79.1 (6.8)	0.1
Sex			
Female	1274 (43.5)	6370 (43.5)	0.0
Male	1654 (56.5)	8270 (56.5)	0.0
Rural residence	246 (8.4)	1050 (7.2)	4.6
Long-term care resident	73 (2.5)	291 (2.0)	3.4
Comorbidity ≤3 y of the index date			
Acute kidney injury	530 (18.1)	2535 (17.3)	2.1
Alcohol use	17 (0.6)	87 (0.6)	0.2
Chronic kidney disease	1677 (57.3)	8724 (59.6)	4.7
Coronary artery disease	382 (13.0)	1802 (12.3)	2.2
Diabetes	2907 (99.3)	14532 (99.3)	0.2
Frailty index			
Robust	193 (6.6)	798 (5.5)	4.8
Prefall	334 (11.4)	1403 (9.6)	6
Frail	350 (12.0)	1436 (9.8)	6.9
Missing	2051 (70.0)	11003 (75.2)	11.5
Heart failure	886 (30.3)	4375 (29.9)	0.8
Hypertension	2737 (93.5)	13763 (94.0)	2.2
Liver disease	132 (4.5)	756 (5.2)	3.1
Prior episode of rhabdomyolysis	99 (3.4)	438 (3.0)	2.2
Stroke or TIA	453 (15.5)	2242 (15.3)	0.4
Hospitalizations ≤1 y prior to index date, mean (SD)	0.82 (1.3)	0.68 (1.2)	11.1
eGFR ≤1 y prior to index date, mean (SD), (mL/min)	54.7 (23.9)	55.8 (22.1)	5.0
Prescribed medications ≤120 d prior to index date			
Antibiotic	914 (31.2)	4730 (32.3)	2.3
Antifungal	36 (1.2)	188 (1.3)	0.5
Diuretic	1367 (46.7)	6977 (47.7)	1.9
Fibric acid derivative	46 (1.6)	219 (1.5)	0.6
Nonsteroidal anti-inflammatory agent	228 (7.8)	1023 (7.0)	3.1
Renin angiotensin system blocker	2052 (70.1)	9895 (67.6)	5.4

Abbreviations: eGFR, estimated glomerular filtration rate; TIA, transient ischemic attack.

An SGLT2i compared with a DPP4i prescription was associated with lower odds of rhabdomyolysis (aOR, 0.75; 95% CI, 0.65-0.87) (Table 2). Odds were similarly lower for empagliflozin and canagliflozin but not significantly lower for dapagliflozin (Table 2).

**Table 2.** Hospital Admission or ED Visit for Rhabdomyolysis by Medication Exposure

Medication exposure group	Adults with rhabdomyolysis admission or ED visit			OR (95% CI)	aOR (95% CI)^b^
	Total, No.	Cases (%) (n = 2928)^a^	Controls (%) (n = 14 640)^a^		
DPP4i	‭15 920	2709 (17.0)	13 211 (83.0)	1 [Reference]	1 [Reference]
SGLT2i	1648	219 (13.3)	1429 (86.7)	0.74 (0.64-0.86)	0.75 (0.65-0.87)
Dapagliflozin	208	30 (14.4)	178 (85.6)	0.81 (0.54-1.20)	0.82 (0.55-1.22)
Empagliflozin	967	129 (13.3)	838 (86.7)	0.75 (0.62-0.90)	0.75 (0.62-0.91)
Canagliflozin	473	60 (12.7)	413 (87.3)	0.69 (0.52-0.91)	0.71 (0.53-0.93)

Abbreviations: aOR, adjusted odds ratio; DPP4i, dipeptidyl peptidase 4 inhibitor; ED, emergency department; SGLT2i, sodium-glucose cotransporter-2 inhibitor.

^a^ Percentages are out of row totals.

^b^ Adjusted for a frailty index and the number of hospitalizations in the 1 year before the index date.

## Discussion

In this case-control study, SGLT2i initiation was associated with a lower risk of rhabdomyolysis compared with DPP4i initiation among older adults receiving long-term statin therapy. As a limitation, unmeasured confounding could explain the association we found. Furthermore, late rhabdomyolysis, or mild disease, may have been missed. Given the cardiovascular and mortality benefits associated with SGLT2i and statin use,^1^ these findings suggest that they may be safely prescribed together when indicated.

## Notes

Supplement 1.
eMethods.
**eTable 1.** Description of ICES databases used in the study **eTable 2.** Variables included in the disease risk score used to match cases and controls **eFigure.** Nested case-control assembly
eReferences.

Supplement 2.
Data Sharing Statement
