# Supplementary material for: Rhabdomyolysis and Sodium-Glucose–Linked Transport Inhibitors in Patients Taking Statins
Source: JAMA Netw Open. 2024 Nov 14;7(11):e2446641. doi: 10.1001/jamanetworkopen.2024.46641 (PMC11565259; doi:10.1001/jamanetworkopen.2024.46641)
Supplement: Supplement 1. — eMethods. eTable 1. Description of ICES databases used in the study eTable 2. Variables included in the disease risk score used to match cases and controls eFigure. Nested case-control assembly eReferences. [file jamanetwopen-e2446641-s001.pdf]

Supplemental Online Content

Harel Z, Jeyakumar N, Smith G, Ray JG, Clemens KK, Juurlink DN. Rhabdomyolysis and sodium-glucose–linked transport inhibitors in patients taking statins. *JAMA Netw Open*. 2024;7(11):e2446641. doi:10.1001/jamanetworkopen.2024.46641

eMethods.

eTable 1. Description of ICES databases used in the study

eTable 2. Variables included in the disease risk score used to match cases and controls

eFigure. Nested case-control assembly

eReferences.

This supplemental material has been provided by the authors to give readers additional information about their work.

© 2024 Harel Z et al. *JAMA Network Open.*

eMethods: Detailed Methods

Study Design and Setting

This population-based nested case-control study examined the association between SGLT2i use and rhabdomyolysis in the whole province of Ontario, Canada, where there is universal healthcare, and free medication coverage for all seniors aged 65 years and older. The study period was between July 1, 2015 and September 23, 2020 (**eFigure 1**).

A nested case-control design was chosen given its advantage in a pharmacoepidemiologic study when an outcome is rare, as in this study.[1] This study encapsulated the era when SGLT2i's were primarily prescribed for the management of diabetes mellitus, rather than for chronic kidney disease or heart failure, based on studies after 2019.[2,3]

All analyses were conducted between January and April 2023, and followed the Strengthening the Reporting of Observational Studies in Epidemiology (STROBE) reporting guideline for cohort studies.[4]

Data sources

We used validated provincial health care administrative databases, housed at ICES, Toronto (**eTable1**). Each patient in Ontario is linked within these databases using a unique encoded identifier. These databases have been extensively used to study drug safety, including the consequences of drug-drug interactions.[5-7]

Identification of patients and outcomes

Included were adults aged ≥ 66 years who were continuously prescribed a statin, based on ≥ 2 prescriptions for a statin in the 210 days before the index date, with the most recent prescription covering the index date. To avoid incomplete medication records, excluded were those aged 65 years, in their first year of eligibility for prescription drug coverage (**eFigure 1**).

From among those on a long-term stating, cases were those who had an emergency department visit or a primary hospitalization diagnosis of rhabdomyolysis. Rhabdomyolysis was defined by ICD-10-CA codes M628 or T796, or a serum creatine kinase value > 5 times the upper limit of normal, both of which are validated measures of this condition.[8,9] The date of the rhabdomyolysis event served as the index date for all analyses.

Each case with rhabdomyolysis was matched to 5 controls, by age +/-1 year, sex, and disease risk score +/-0.2 standard deviations (SD). The disease risk score was derived by constructing a multivariable regression model that included potential predictors of rhabdomyolysis, including demographic characteristics, comorbidities, markers of health services utilization and medications (**eTable 2**). To mirror the

© 2024 Harel Z et al. *JAMA Network Open.*

temporal distribution of the index date among the cases, each control was randomly assigned an index date.

Exposure

The exposure of interest was the prescription of an SGLT2i (canagliflozin, dapagliflozin, or empagliflozin) ≤ 100 days of receiving a statin prescription. The comparator exposure was a prescription of a dipeptidyl peptidase 4 inhibitor (DPP4i) ≤ 100 days of receiving a statin prescription. An SGLT2i or DDP4i could be prescribed as a single agent, or combined with another diabetes medication. An individual could also have multiple at-risk intervals of SGLT2i or DPP4i use; however, each interval could not be preceded by a prescription for the other drug type or a subclass of each exposure in the 100 days prior to the index date. The time period of up to 100 days after initiation of the exposure drug was chosen as it is hypothesized that the interaction of an SGLT2i and a statin occurs by competitive inhibition of one or more transporters,[10] increasing the blood concentration of the statin, and thus, heightening the risk for rhabdomyolysis. Based on this mechanism, the effect of the interaction between statins and SGLT2i would be expected to occur after a relatively short duration of time (e.g., within 100 days).

DDP4i's were selected as the comparator exposure medication, for several reasons. First like SGLT2i, they were frequently used as second-line therapy for type 2 diabetes during the study era. Second, they are not known to be associated with rhabdomyolysis. Third, they are safe to use in individuals with comorbid conditions, including chronic kidney disease (CKD) and heart failure, while other agents, such as sulfonylureas and biguanides were relatively contraindicated in these conditions due to side effects.[11]

Statistical analysis

Patient characteristics were summarized using descriptive statistics. Cases and controls were compared on their characteristics using standardized differences, with a value greater than 0.10 considered to be a meaningful difference.

Conditional logistic regression generated adjusted odds ratios (OR) and 95% CI for the association between SGLT2i exposure and rhabdomyolysis, using DPP4i as the comparator. Secondary analysis explored the association between SGLT2i sub-type and rhabdomyolysis. Given imbalances between cases and controls in the frailty index and number of hospitalizations within 1 year before the index date, odd ratios were adjusted for these two covariates.

Analyses were performed at ICES using SAS, version 9.4 (SAS Institute Inc).

eTable 1: Description of the ICES databases used in the study.

Dataset name	Description
Canadian Institute for Health Information Discharge Abstract Database (CIHI-DAD)	Contains administrative, clinical (diagnoses and procedures/interventions), demographic, and administrative information for all admissions to acute care hospitals in Ontario. Diagnostic codes are captured using the International Statistical Classification of Diseases and Related Health Problems, 10th Revision, Canada (ICD-10-CA) coding system and interventions (up to 20 on a given DAD record) are captured using the Canadian Classification of Health Interventions (CCI) coding system.
Ontario Drug Benefit (OBD)	Contains all outpatient prescription medications dispensed to Ontarians over the age of 65, and has an accuracy exceeding 99% in comparison to the reference standard of pharmacy chart review.[12]
National Ambulatory Care Reporting System (NACRS)	Contains administrative, clinical (diagnoses and procedures), demographic, and administrative information for all Ontarian patient visits made to hospital- and community-based ambulatory care centres (emergency departments, day surgery units, hemodialysis units, and cancer care clinics).
Ontario Health Insurance Plan (OHIP) Claims History Database	Contains information on inpatient and outpatient services provided to Ontario residents eligible for the province's publicly funded health insurance system by fee-for-service health care practitioners (primarily physicians) and "shadow billings" for those paid through non-fee-for-service payment plans.
Ontario Laboratories Information System (OLIS)	Contains information regarding lab test orders and results from hospitals, community labs, and public health labs across Ontario.
Registered Persons Database (RPDB)	Provides basic demographic information for those issued an Ontario health insurance number, and indicates the time periods for which an individual was eligible to receive publicly funded health insurance benefits and the best-known postal code for each registrant on July 1st of each year.

© 2024 Harel Z et al. *JAMA Network Open.*

eTable 2: Variables included in the disease risk score used to match cases and controls.

**Demographics**
Age
Sex
Income quintile
Rural residence
Fiscal index year
Long term care status
**Comorbid conditions in the preceding 3 years**
Acute kidney injury
Alcoholism
Chronic kidney disease
Coronary artery disease
Diabetes
Frailty index (robust, pre-frail, frail, missing)
Heart failure
HIV
Hypertension
Hypothyroidism
Drug dependence or tobacco use
Liver disease
Previous hospital encounter with rhabdomyolysis
Peripheral vascular disease
Stroke/TIA
**Health care utilization within the preceding 1 year**
Hospitalization episodes
**Medication use within the preceding 120 days**
Antibiotics (not including 3A4 inhibitors)
Antifungals (not including3A4 inhibitors)
Bile acid sequestrants
Colchicine
Diuretics
Fibric acid derivatives
NSAIDs
RAAS blockers
Thyroxine
**Laboratory markers within the preceding 365 days**
Baseline estimated glomerular filtration rate

© 2024 Harel Z et al. *JAMA Network Open.*

eFigure 1: Nested case-control assembly.

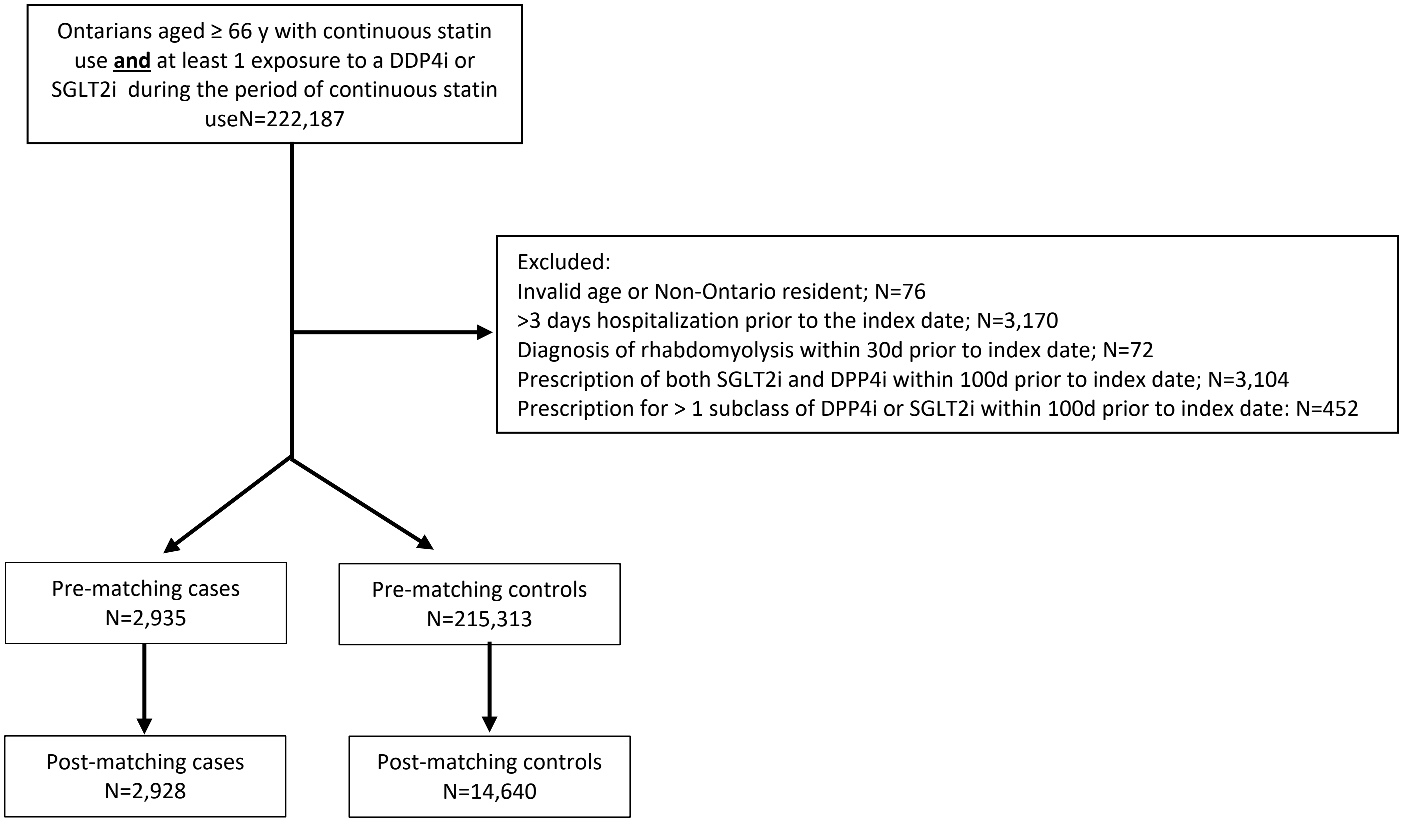

© 2024 Harel Z et al. *JAMA Network Open.*

eReferences

1. DiPietro NA. Methods in epidemiology: observational study designs. Pharmacotherapy 2010;30:973-84.
2. Heerspink HJL, Stefánsson BV, Correa-Rotter R, et al. Dapagliflozin in Patients with Chronic Kidney Disease. N Engl J Med 2020;383:1436-46.
3. Packer M, Anker SD, Butler J, et al. Cardiovascular and Renal Outcomes with Empagliflozin in Heart Failure. N Engl J Med 2020;383:1413-24.
4. von Elm E, Altman DG, Egger M, Pocock SJ, Gøtzsche PC, Vandenbroucke JP. Strengthening the Reporting of Observational Studies in Epidemiology (STROBE) statement: guidelines for reporting observational studies. Bmj 2007;335:806-8.
5. Harel Z, McArthur E, Jeyakumar N, et al. The Risk of Acute Kidney Injury with Oral Anticoagulants in Elderly Adults with Atrial Fibrillation. Clin J Am Soc Nephrol 2021;16:1470-9.
6. Lee EY, Gomes T, Drucker AM, et al. Oral Antibiotics and Risk of Serious Cutaneous Adverse Drug Reactions. Jama 2024;332:730-7.
7. Ray JG, Harel Z, Gilbert RE, Wald R, Berger H, Park AL. Preconception SGLT2 or DPP4 inhibitor use and adverse pregnancy outcomes. Diabetes Res Clin Pract 2023;205:110946.
8. Chavez LO, Leon M, Einav S, Varon J. Beyond muscle destruction: a systematic review of rhabdomyolysis for clinical practice. Crit Care 2016;20:135.
9. Patel AM, Shariff S, Bailey DG, et al. Statin toxicity from macrolide antibiotic coprescription: a population-based cohort study. Ann Intern Med 2013;158:869-76.
10. Vallon V. State-of-the-art-review Mechanisms of action of SGLT2 inhibitors and clinical implications. Am J Hypertens 2024.
11. Davies MJ, Aroda VR, Collins BS, et al. Management of Hyperglycemia in Type 2 Diabetes, 2022. A Consensus Report by the American Diabetes Association (ADA) and the European Association for the Study of Diabetes (EASD). Diabetes Care 2022;45:2753-86.
12. Levy AR, O'Brien BJ, Sellors C, Grootendorst P, Willison D. Coding accuracy of administrative drug claims in the Ontario Drug Benefit database. Can J Clin Pharmacol 2003;10:67-71.

© 2024 Harel Z et al. *JAMA Network Open.*
