# Supplementary material for: Rhabdomyolysis and Sodium-Glucose–Linked Transport Inhibitors in Patients Taking Statins
Source: JAMA Netw Open. 2024 Nov 14;7(11):e2446641. doi: 10.1001/jamanetworkopen.2024.46641 (PMC11565259; doi:10.1001/jamanetworkopen.2024.46641)
Supplement: Supplement 2. — Data Sharing Statement [file jamanetwopen-e2446641-s002.pdf]

Data Sharing Statement

Harel. Rhabdomyolysis and Sodium-Glucose–Linked Transport Inhibitors in Patients Taking Statins. *JAMA Netw Open*. Published online November 14, 2024. doi:10.1001/jamanetworkopen.2024.46641

Data
Data available: No
